# Supplementary material for: Adaptive Neural Reorganization Enables Real-Time Finger-Level Robotic Control in BCI-Naïve Stroke Survivors
Source: bioRxiv. 2026 Jun 18:2026.06.15.732267. Preprint. [Version 1] doi: 10.64898/2026.06.15.732267 (PMC13307948; doi:10.64898/2026.06.15.732267)
Supplement: Supplement 1 [file NIHPP2026.06.15.732267v1-supplement-1.pdf]

bioRxiv preprint doi: https://doi.org/10.64898/2026.06.15.732267; this version posted June 18, 2026. The copyright holder for this preprint (which was not certified by peer review) is the author/funder, who has granted bioRxiv a license to display the preprint in perpetuity. It is made available under a CC-BY 4.0 International license.

# Supporting Information

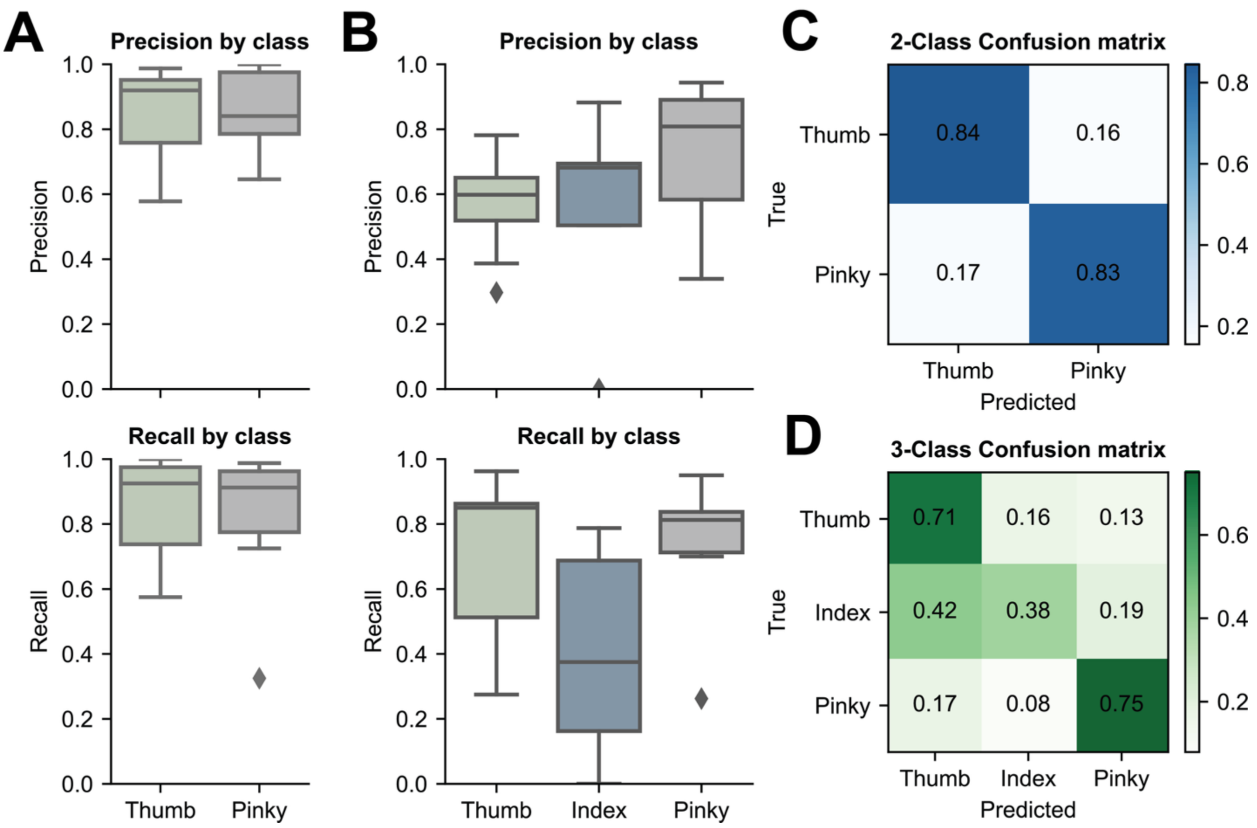


**S1 Fig. Online motor imagery (MI)–based robotic finger control performance (*n* = 9).** (**A** and **B**) Group-level precision and recall of 2-finger (**A**) and 3-finger (**B**) decoding. The center lines indicate the median value. The boxes extend from the lower quartile to the upper quartile, and the lines indicate 1.5 times the interquartile range. Diamonds indicate outliers that are more than 1.5 times the interquartile range above the third quartile or below the first quartile. (**C** and **D**) Confusion matrices for 2-finger (**C**) and 3-finger (**D**) online decoding results. The values represent the proportion of predictions for each true label classified as each predicted label.

bioRxiv preprint doi: https://doi.org/10.64898/2026.06.15.732267; this version posted June 18, 2026. The copyright holder for this preprint (which was not certified by peer review) is the author/funder, who has granted bioRxiv a license to display the preprint in perpetuity. It is made available under a CC-BY 4.0 International license.

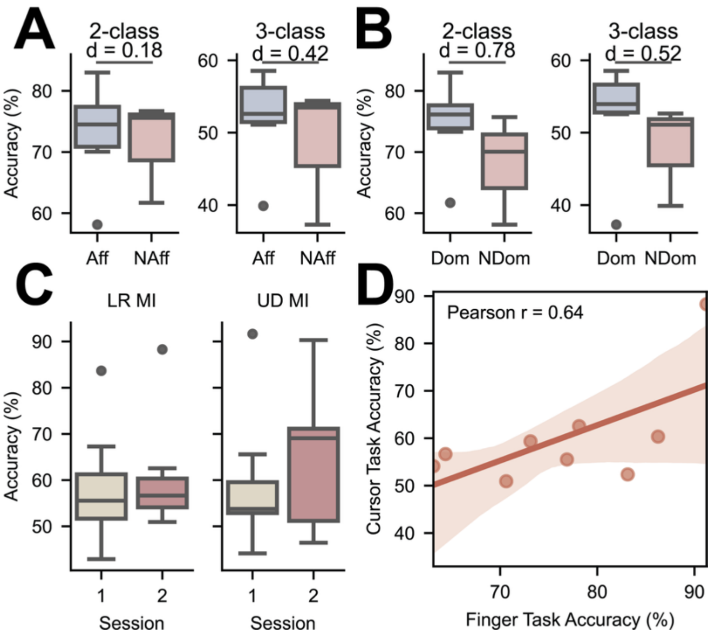


**S2 Fig. Subgroup analysis of online finger MI control performance and its relationship to limb-level MI decoding.** (**A**) Comparison of 2-finger and 3-finger online decoding performance between stroke survivors who performed MI control using their affected hand (*n* = 6) and those using their non-affected hand (*n* = 3). (**B**) Comparison of 2-finger and 3-finger online decoding performance between stroke survivors who performed MI control using their dominant hand (*n* = 6) and those using their non-dominant hand (*n* = 3). (**C**) Online performance in the limb-level cursor MI training task across two sessions. (**D**) Correlation between online finger-level MI decoding accuracy and cursor-task performance (*n* = 9). The solid line represents the linear regression fit, and the shaded region denotes the 95% confidence interval.

bioRxiv preprint doi: https://doi.org/10.64898/2026.06.15.732267; this version posted June 18, 2026. The copyright holder for this preprint (which was not certified by peer review) is the author/funder, who has granted bioRxiv a license to display the preprint in perpetuity. It is made available under a CC-BY 4.0 International license.

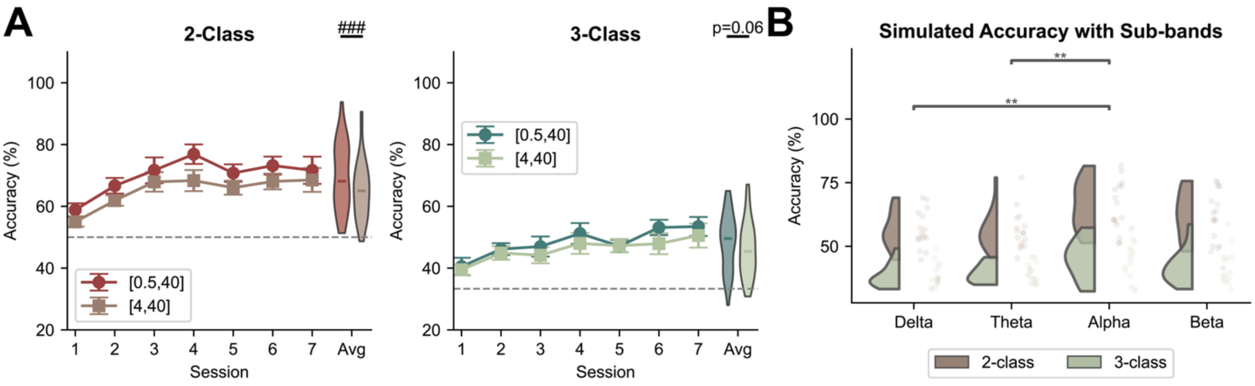


**S3 Fig. Offline analysis of low-frequency contributions.** (**A**) Simulated offline base decoding accuracy using EEG inputs filtered at 0.5–40 Hz and 4–40 Hz for 2-finger and 3-finger MI across seven online sessions ($n = 9$). The grey dashed line represents the chance level, and error bars indicate the standard error. The performance distribution across all sessions is shown on the right. The center lines indicate the median value. The boxes extend from the lower quartile to the upper quartile, and the lines indicate 1.5 times the interquartile range. Statistical analysis was performed using a two-way repeated-measures ANOVA with main effects of session and bandpass filtering setting. Significant main effects of bandpass are indicated (### if $p < 0.001$). (**B**) Simulated decoding accuracy using EEG signals filtered into individual frequency bands for the healthy control group (delta, theta, alpha, and beta) ($n = 16$). Statistical significance was observed for the main effect of frequency bands in a two-way ANOVA. Significance stars indicate post hoc pairwise comparison results using an FDR-corrected two-tailed Wilcoxon signed-rank test (** if $p < 0.01$).

bioRxiv preprint doi: https://doi.org/10.64898/2026.06.15.732267; this version posted June 18, 2026. The copyright holder for this preprint (which was not certified by peer review) is the author/funder, who has granted bioRxiv a license to display the preprint in perpetuity. It is made available under a CC-BY 4.0 International license.

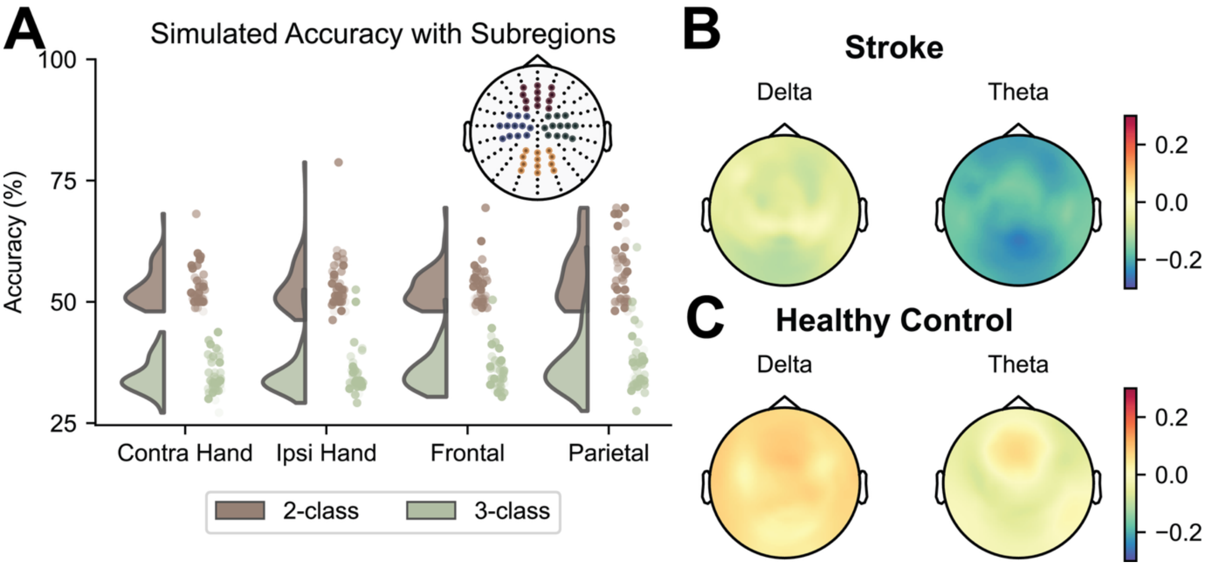


**S4 Fig. Offline analysis of cortical subregion contributions and task-induced electrophysiologies. (A)** Simulated decoding accuracy using EEG signals within individual cortical subregions (*n* = 9). The channels within the contralateral hand (Contra Hand), ipsilateral hand (Ipsi Hand), frontal, and parietal regions are colored in blue, green, red, and yellow, respectively. **(B** and **C)** Group-averaged topographical maps of delta-band (0.5-4 Hz) and theta-band (4-8 Hz) ERD during online MI control for the stroke group (**B,** *n* = 9) and the healthy control group (**C,** *n* = 16).

bioRxiv preprint doi: https://doi.org/10.64898/2026.06.15.732267; this version posted June 18, 2026. The copyright holder for this preprint (which was not certified by peer review) is the author/funder, who has granted bioRxiv a license to display the preprint in perpetuity. It is made available under a CC-BY 4.0 International license.

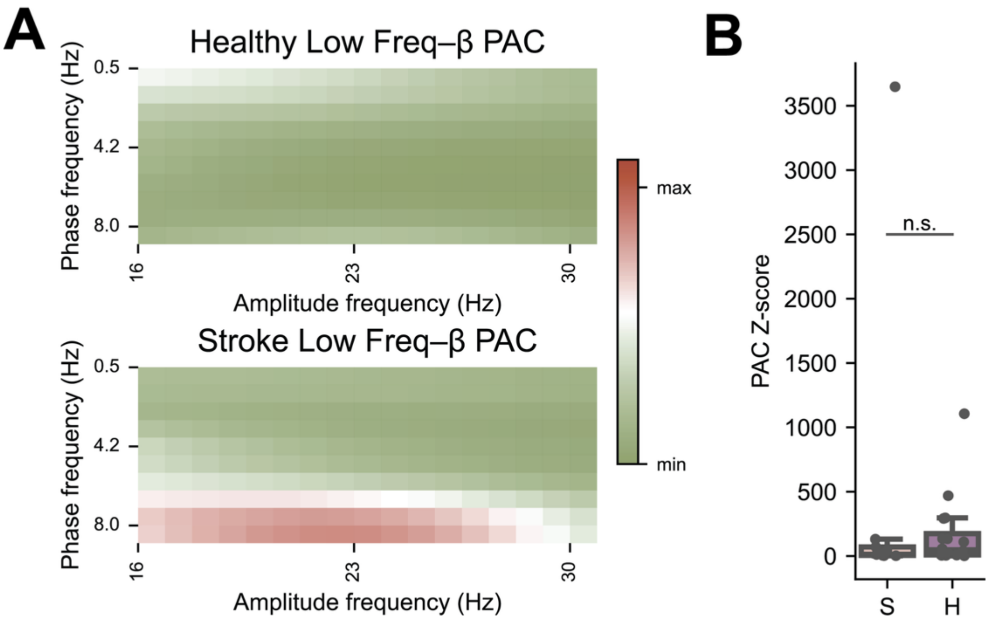


**S5 Fig. Cross-frequency correlation in stroke survivors.** (**A**) The phase-amplitude coupling (PAC) of the stroke and healthy group between low frequency activities (0.5 – 8 Hz) and beta activities over the contralateral sensorimotor cortex (the ipsilesional sensorimotor cortex for stroke survivors). PAC values were normalized to the maximum value observed across the two groups for visualization. (**B**) Comparison of PAC z-scores between groups. Z-scores were computed based on surrogate distributions generated using 200 permutations. Between-group differences were assessed using two-tailed Mann–Whitney U tests (n.s. if no statistical significance is found).

bioRxiv preprint doi: https://doi.org/10.64898/2026.06.15.732267; this version posted June 18, 2026. The copyright holder for this preprint (which was not certified by peer review) is the author/funder, who has granted bioRxiv a license to display the preprint in perpetuity. It is made available under a CC-BY 4.0 International license.

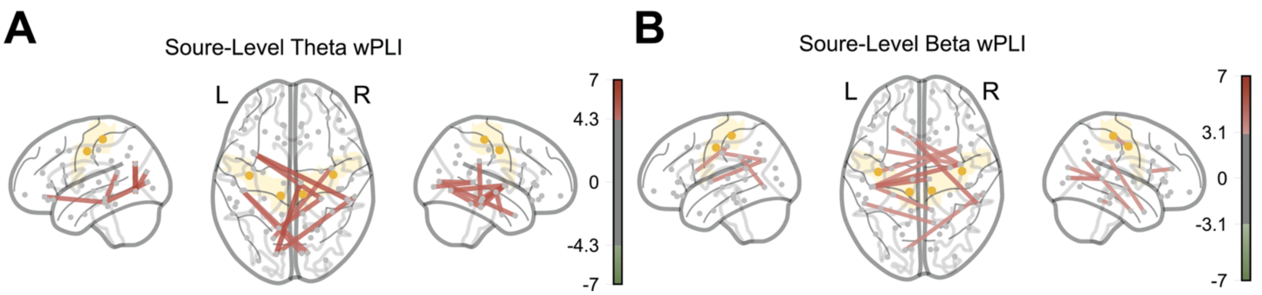


**S6 Fig. Group-level differences in theta-band (A) and beta-band (B) weighted Phase Lag Index (wPLI) at the source level, expressed as z-scores relative to healthy controls.** Functional connections between the atlas with absolute z-scores exceeding the 99th percentile threshold are displayed, with edge color and thickness indicating the sign and magnitude of the z-score.
